# Assessment of radiopharmaceutical retention for vascular access ports using positron emission tomography imaging

**DOI:** 10.1002/acm2.12196

**Published:** 2017-10-05

**Authors:** Michael S. Gossman, Huaiyu Zheng, John G. Evans, Junling Li, Chin K. Ng

**Affiliations:** ^1^ Regulation Directive Medical Physics Russell KY USA; ^2^ Department of Radiology University of Louisville School of Medicine Louisville KY USA; ^3^ Bard Peripheral Vascular Salt Lake City UT USA

**Keywords:** FDG, Na^18^F, PET, tracer retention, vascular access

## Abstract

**Purpose:**

The purpose of this study was to resolve the issue of whether various generations of CR Bard peripheral vascular access ports and catheters are prone to retain PET radiopharmaceuticals. The study evaluates the residual radioactivity remaining following injection for two PET radiopharmaceuticals currently used extensively in the clinic, FDG and Na^18^F.

**Methods:**

FDG was purchased from a local cyclotron facility and Na^18^F was prepared in‐house. Three generations of currently marketed vascular access ports were tested. A total of five (*n* = 5) of each model was tested. Radiopharmaceutical of 2–3 mCi of each was injected into each port and flushed with 10, 30, 60, and 120 ml of saline. MicroPET scans were performed after each flush to detect the residual radioactivity on each port. A dose calibrator was used to detect the retention of radioactivity after each flush.

**Results:**

Radioactivity retention for all vascular port models measured by microPET imaging was similar for both FDG and Na^18^F, with less than 1% residual activity following a 10 ml saline flush. Based on the microPET images, all the subsequent flushes of 30, 60, and 120 ml were also considered. Dose calibrator activity measurements validated microPET measurements as negligible for all the ports, even with the first 10 ml flush.

**Conclusions:**

MicroPET imaging was more sensitive than the dose calibrator in determining the radioactivity retention of the vascular access ports from CR Bard. These ports may be used for the injection of FDG and Na^18^F to track glucose metabolism and bone uptake with PET imaging. It is recommended to apply at least a 10 ml flush after radiopharmaceutical administration, to reduce residual activity to baseline levels.

## 1 INTRODUCTION

Vascular access ports have been used for decades as a means to deliver local anesthetic, chemotherapeutic agents, and prescription drugs as well as to extract blood for testing.^1^, ^2^ Use of vascular access ports relieve the number of needle sticks, while also giving patients a sense of freedom in not having to be reminded constantly of their illness.^3^ The position of the port is generally in the upper chest region. With a minor surgery technique, surgeons can quickly insert the catheter of the port into a vein, while subcutaneously positioning the port body such that the septum is immediately under the skin surface.

Computerized tomography examinations are commonly performed with the intravenous use of iodinated contrast media, either as a fundamental part of the examination or to improve soft‐tissue distinction.^4^ Port systems allow for liquids to be injected into the bloodstream at a higher rate than by peripherally inserted central catheter injection. The use of vascular access ports is considered by many physicians to be a significant alternative to prevent potentially damaging attempts at percutaneous indwelling access. Physicians must weigh the relative risks and benefits of using a vascular access port for power injection of contrast material, as they will have a clearer knowledge of the relative importance of contrast enhancement for accurate physiological and anatomical interpretation.^4^

Positron emission tomography (PET) is a noninvasive diagnostic tool that similarly provides tomographic images, while enabling observation of perfusion, cell viability, proliferation, and metabolic activity in tissues. The use of miniature PET imaging systems for molecular imaging in preclinical research has been shown to be a valuable tool and is gaining increased use in many fields including drug development.^5^ There have been conflicting anecdotal comments on administration of PET radiotracers through ports with regard to activity retention within the port body and the attached catheter following injection. The majority of the risk in using a port is whether the radiopharmaceutical is taken up by the materials of the device that may result in residual image artifacts. Undesirable contrast, resolution, and artifacts can lead to a potential error for physiological PET tracer uptake errors in diagnosis.^6^, ^7^

An international team has already investigated similar issues, leading up to the need for this study. They looked at hydrodynamics and temperature as key parameters for the efficiency of a vascular access device to rid contrast media directly. The group evaluated six devices (*n* = 6) and indicated using a Mann–Whitney U‐test that port cavities are incompletely rinsed after three consecutive flushes of 10 ml saline solution for a net 30 ml flush.^8^

Here, we explore the ramifications of introducing various port and catheter designs as well as two commonly used radiotracers using PET imaging: FDG and Na^18^F. Similarly, testing of a total of five devices (*n* = 5) of each port model were explored. Results from consecutive flushes of injected saline are described for each port and tracer type.

## 2 MATERIALS AND METHODS

Radioactive fluoride (^18^F) and ^18^F‐fluorodeoxyglucose (FDG) was first purchased from PETNET Solutions, Inc. (Louisville, KY, USA). Radiolabeled ^18^F‐Sodium Fluoride (Na^18^F) was then prepared from the acquired ^18^F in‐house as described in the literature.^9^, ^10^ Briefly, the process began with ^18^F‐fluoride and 0.1 ml H^18^O diluted with 5 ml sterile water and passed through a cation exchange (in the form of H^+^) cartridge connected to a Waters Corporation Sep‐Pak Accell Plus QMA cartridge (Milford, MA, USA). The cation exchange cartridge was then removed. The QMA cartridge was rinsed with 10 ml sterile water and air dried. Finally, Na^18^F was eluted with 5–10 ml saline and passed through a 0.2 μm filter to provide the end product for imaging. The yield for Na^18^F was better than 90%.

The imaging system of choice for this study was a Siemens R4 MicroPET scanner (Knoxville, TN, USA). Images were reconstructed using an OSEM2D algorithm and analyzed with ASIPro software. The dedicated third‐generation PET scanner was capable of obtaining millimeter‐level high‐spatial resolution at the center of the field of view. Calibration and quality assurance testing were performed prior to any acquisition. To accompany PET activity detection, radioactivity was also measured using a dose calibrator (Biodex Atomlab 500).

Various vascular access ports were evaluated separately and identically. Ports were supplied by CR Bard (Salt Lake City, UT, USA). Three port designs currently marketed were selected for testing: PowerPort^®^ isp (Model 1708560) titanium device with a 8.0 Fr Groshong^®^ single‐lumen venous catheter, PowerPort^®^ isp M.R.I. (Model 1809660) acetal device with a 9.6 Fr silicone open‐ended single‐lumen venous catheter, and PowerPort^®^ ClearVUE^®^ (Model 1618000) slim poly‐ether‐ether‐ketone device with a 8.0 Fr polyurethane open‐ended single‐lumen venous catheter. The imaging protocol is outlined in Table 1.

**Table 1:** Imaging protocol

Background scan	PET scan saline‐filled port and catheter for 10 min as background control
Radioisotope	Measure received radioisotope FDG or Na^18^F in dose calibrator and inject 2–3 mCi of FDG or Na^18^F into all five ports and catheter sets for first model. Measure activity contained in each port and catheter set in dose calibrator separately
Sample preparation	Isolate one port and catheters set as a contrast control and mount; isolate the remaining port and catheters set as the sample, and then mount. Stack the mounted sets with the contrast control underneath and position on the PET scanner
Control images	Obtain scans for positive and negative control
Sample image #1	Flush the sample with 10 ml of saline and scan those three ports and catheters on MicroPET for 10 min
Sample image #2	Measure received radioisotope FDG or Na^18^F in dose calibrator. Flush the sample with 30 ml of saline and scan those three ports and catheters on MicroPET for 10 min
Sample image #3	Measure received radioisotope FDG or Na^18^F in dose calibrator. Flush the sample with 60 ml of saline and scan those three ports and catheters on MicroPET for 10 min
Sample image #4	Measure received radioisotope FDG or Na^18^F in dose calibrator. Flush the sample with 120 ml of saline and scan those three ports and catheters on MicroPET for 10 min
Repeat	Repeat process from beginning for two other models of port and catheter sets for the same contrast, and then repeat all with the other contrast

**Figure 1:**
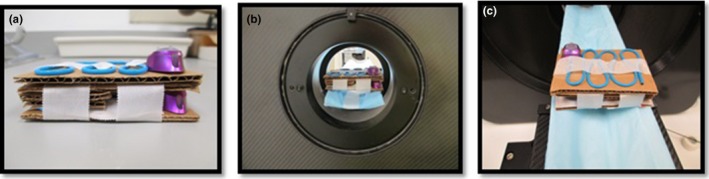
Set‐up for MicroPET scan acquisition: (a) Two ports/catheters sitting on the laboratory bench with the positive control at the bottom, (b) The same two ports/catheters sitting inside the MicroPET scanner, and (c) The same two ports/catheters sitting on the MicroPET scanner bed.

Each port and catheter pairing was primed with 2 ml saline and heated at 37°C for 24 hr prior to use. Five sets (*n* = 5) for each of the three port designs were tested, using both FDG and Na^18^F, resulting in a total of 30 ports used in this study. For each type, one was evaluated as a negative control. Ports were then subject to injection of 2–3 mCi (74–111 MBq) of FDG or Na^18^F, respectively, where they served as positive controls. The ports were individually and identically taped to a minified cardboard couch and positioned to best fit within the field of view for the scanner (Fig. 1).

There was no time lag in between flushes with imaging. Volumes were introduced cumulatively. For example, the first flush was 10 ml with imaging, followed by an additional 20 ml to additively arrive at 30 ml for the second level with imaging, and so on to 60 and 120 ml. A PET scan was then performed following each repetitive flush. Regions‐of‐interest (ROIs) were manually drawn over the images using ASIPro software that is used specifically with the microPET‐R4 scanner. From these ROIs, the mean voxel values of the radioactive decay counts were determined. The net activity of each port and catheter type was obtained by summation of activity in 20 consecutive coronal slices. Retention rates were calculated as 100% x (Counts in each flushed sample–Counts of negative control)/(Total counts of positive control). The residual radioactivity for each sample was also measured using a well‐counter detector system to further confirm the PET results.

In order to understand whether dead‐time and cross‐talk from adjacent slices played a significant role in our measurements, tubing was prepared and filled with 1.65 mCi of FDG in 1.2 ml volume. It was then put on top of another piece of tubing without being filled with FDG inside the field of view of the microPET scanner. MicroPET images for this entire assembly were taken serially every hour for 7 hr.

An analysis of variance statistical model was used to compute one‐way test *P*‐values.^11^ Consistent with standard usage, only *P*‐values less than *α* = 0.05 were considered significant. Results validate the hypothesis that specific volumes of saline are necessary to remove residual activity of either FDG or Na^18^F in each of the port models considered.

## 3 RESULTS

**Figure 2:**
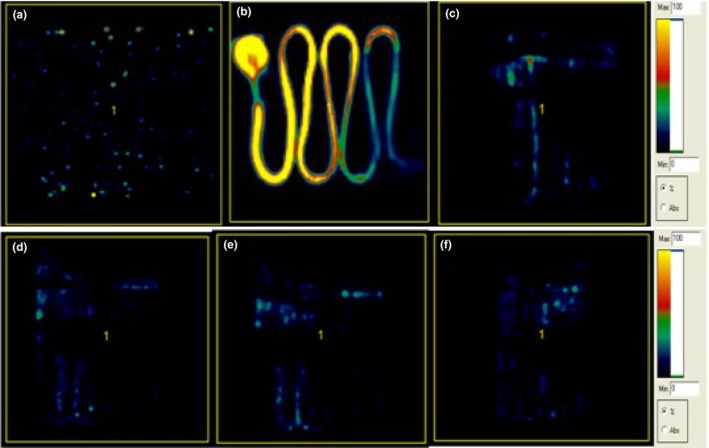
Representative FDG PET images for CR Bard PowerPort^®^ isp (Model 1708560): (a) Saline only, (b) Positive FDG control, (c) After 10 ml of saline flush, (d) After 30 ml of saline flush, (e) After 60 ml of saline flush, and (f) After 120 ml of saline flush.

**Figure 3:**
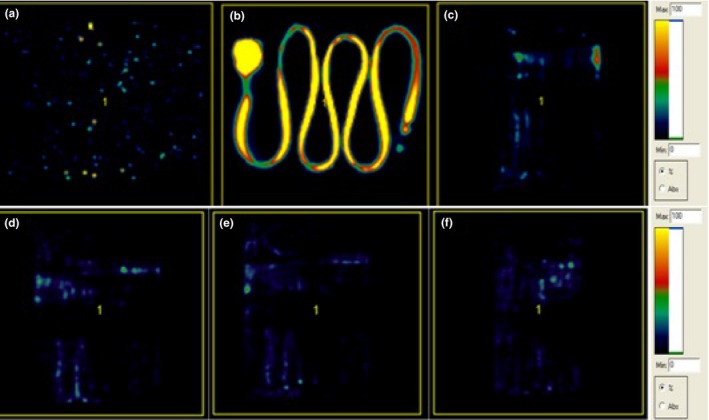
Representative Na^18^F PET images for CR Bard PowerPort^®^ isp (Model 1708560): (a) Saline only, (b) Positive Na^18^F control, (c) After 10 ml of saline flush, (d) After 30 ml of saline flush, (e) After 60 ml of saline flush, and (f) After 120 ml of saline flush.

The retention rates for different port models based on PET quantification are shown in Table 2 for FDG and Table 3 for Na^18^F. Figures 2 and 3 showed some representative microPET images obtained specifically for the CR Bard PowerPort^®^ isp (Model 1708560) in different conditions. For the FDG experiment, the percentage (%) retention for PowerPort^®^ isp (model 1708560) was reduced from 0.80% to 0.73% over the flushing volume between 10 and 120 ml. Retention for PowerPort^®^ isp M.R.I (model 1809660) was reduced from 0.66% to 0.61%, while for PowerPort^®^ ClearVUE^®^ (model 1618000) it was reduced from 0.66% to 0.54% when the flushing volume went from 10 to 120 ml. The average percentage retention for all three models after a 10 ml saline flush was 0.71 ± 0.22%. For Na^18^F, a similar trend was observed in all three port models. The percentage retention did not show any significant difference among all three models, with an average of 0.69 ± 0.26%. Moreover, there was no remarkable benefit to flushing more than 10 ml, although some reduction was noted. The average change for ports evaluated with either contrast agent between 10 and 120 ml was a mere 0.10 ± 0.10% on average. For both tracers, radioactivity for all port models measured by the well‐counter was close to background after flushing with 10 ml of saline.

**Table 2:** Percentage retention of FDG for different CR Bard port system models (*n* = 5)

Model	10 ml	30 ml	60 ml	120 ml
1708560	0.80 ± 0.15%	0.73 ± 0.03%	0.72 ± 0.04%	0.73 ± 0.03%
1809660	0.66 ± 0.12%	0.63 ± 0.11%	0.60 ± 0.13%	0.61 ± 0.13%
1618000	0.66 ± 0.39%	0.54 ± 0.16%	0.53 ± 0.18%	0.54 ± 0.17%

**Table 3:** Percentage retention of Na^18^F for different CR Bard port system models (*n* = 5)

Model	10 ml	30 ml	60 ml	120 ml
1708560	0.81 ± 0.22%	0.73 ± 0.14%	0.67 ± 0.10%	0.64 ± 0.08%
1809660	0.66 ± 0.31%	0.57 ± 0.19%	0.55 ± 0.18%	0.55 ± 0.17%
1618000	0.60 ± 0.24%	0.53 ± 0.23%	0.50 ± 0.25%	0.50 ± 0.24%

The spill‐over of counts from the chosen PET image slice to adjacent slices were expectedly of the same order of magnitude as our retention measurements.

Statistical *P*‐values for all models of ports tested with both FDG and Na^18^F are reported in Tables 2 and 3. Measured activities were deemed not statistically different from one another when the *P*‐value >0.05. All *P*‐values were estimated to be larger than 0.05 among all the groups with different flushing amount for all three port models. From this data, it was concluded that only a 10 ml saline flush was required following the administration of either FDG or Na^18^F.

## 4 DISCUSSION

In this study, three types of CR Bard vascular access port types were evaluated and compared for their inherent ability to retain or release radioactive contrast agents used for PET imaging. We note that clinically, there could be instances of retention in vascular ports that are potentially related to catheter complications such as thrombosis or the growth of a fibrin sheath over the catheter. Still, the port and catheter should be flushed with 10 ml of sterile saline before and after each use on patients.^1^ Over 99% of radiotracers were removed after 10 ml flush in these tests, regardless of whether the tracer was FDG or Na^18^F. Each port‐catheter‐contrast combination test resulted in activities indistinguishable from background as measured by the well‐counter after only a single flush with 10 ml saline. Tracing of residual activity were only exhibited on some scan slices under maximum resolution within the microPET ASIPro^®^ software (Fig. 2). Residual activity was concluded to be the result of a combination of both (a) surface tension: the force of adhesion of FDG or Na^18^F as it comes in contact with a different molecule that is either liquid or solid (the port system) and (b) capillary action: the ability of the liquid molecule (the contrast) to flow in a finite sized passage (the port system) without the assistance of external forces such as gravity. Residual activity levels are always present in PET scans when used clinically. Remnant activity, less than 1% of the administered amount, was therefore of no negative consequence for the study.

In order to evaluate if there might be a chance of having activity contamination, which could affect the ROI values, ports were re‐taped onto the cardboard after each flush prior to PET imaging. We also considered the possibility of slice numbers within the field of view being different or angled. Care was applied so as to duplicate alignment of the cardboard couch and to determine retention in the same couch position for all measurements, using the same technique.

**Figure 4:**
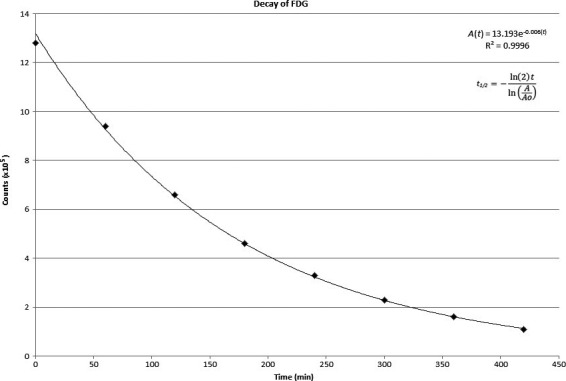
Time activity curve generated from a series of microPET images taken every hour for 7 hr. 1.65 mCi of FDG in 1.2 ml filled up the tubing.

Dead‐time was also an unlikely contributing factor in this process. The measured decay over time for ^18^F is shown in Fig. 4. The known half‐life is 109.8 min. The nominal measured value was roughly the same by measurement. The decay plot validates the quantitative results of the microPET R4 scanner, since the half‐life of ^18^F could be accurately measured. Therefore, the percentage retention estimated by microPET imaging was reliable.

The background level for an empty piece of tubing sitting next to another piece of tubing filled with FDG was estimated to be 1.61 ± 0.06%. This result was larger than any numbers obtained from retention measurements. The larger percentage was probably due to the fact that the gap used for the assessment of dead‐time was smaller than that used for the percentage retention experiment. Again, all the ports did not contain any significant amount of residual radioactivity as confirmed by the dose calibrator measurements.

Results obtained from FDG and Na^18^F testing are not necessarily transferable to other radiotracers.^12^, ^13^ Further investigation is needed to examine how other radiotracers interact with various vascular port and PICC line systems. Since there are many different intravascular device manufacturers and models, as well as other tracer radionuclides in use, no single result from one of these port designs can be assumed to depict the inherent physical characteristics of ports from another manufacturer or with a different tracer. We caution that similar testing could potentially reveal the presence of above background remaining activity with only a single 10 ml saline flush. If remarkable activities remain even after saline flushing, such contrast could give rise to image artifacts that affect diagnostic reading, in the form of false positives or negatives, and even further affect treatment options for patients.^14^, ^15^

## 5 CONCLUSION

We examined three of the most common vascular access port systems from CR Bard *ex vivo* in order to characterize the ability of each port in retaining FDG and Na^18^F. Both PET radiotracers are routinely used in the clinic. The PET scan analysis and dose calibrator assays revealed that no significant residual radioactivity remained after only a 10 ml flush. No remarkable benefit was identified when flushing more than 10 ml. The average change for ports evaluated with either contrast agent between 10 and 120 ml was mere 0.1%. These evaluated vascular access ports from CR Bard may be used for the injection of FDG and Na^18^F used for PET imaging.

## CONFLICTS OF INTEREST

The authors have no relevant financial interests in the manuscript and no other potential conflicts of interest to disclose.
